# Synthesis and drag reduction properties of a hydrophobically associative polymer containing ultra-long side chains

**DOI:** 10.1186/s13065-023-00968-5

**Published:** 2023-06-05

**Authors:** Xianwu Jing, Youquan Liu, Wanwei Zhao, Junhong Pu

**Affiliations:** 1 Research Institute of Natural Gas Technology, Southwest Oil and Gas Field Company, China National Petroleum Corporation (China) CN, Chengdu, 610213 Sichuan People’s Republic of China; 2 Shale Gas Evaluation and Exploitation Key Laboratory of Sichuan Province, Sichuan Provincial Department of Science and Technology, Chengdu, 610051 Sichuan People’s Republic of China; 3 Engineering Technology Department, Southwest Oil and Gas Field Company, China National Petroleum Corporation (China) CN, Chengdu, 610081 Sichuan People’s Republic of China

**Keywords:** Ultra-long hydrophobic monomer, Drag reducer, Friction, Drag reduction rate, Cryo-TEM

## Abstract

Different from common hydrophobic associative polymers, a new hydrophobic associative polyacrylamide (HAPAM) with ultra-long side chains was synthesized and aimed to be used as drag reducer in this work. Firstly, a water-soluble hydrophobic monomer (named AT114) was obtained by alcoholysis reaction with acryloyl chloride and triton 114, then the drag reducer was obtained by radical copolymerization of AM, AMPS and AT114. The structures of AT114 and drag reducer were characterized by IR and NMR. Slick water was obtained by dissolving a small amount drag reducer in water. Although the viscosity of slick water varied greatly in fresh water and brine, the drag reduction rate always remained at a high level when flowing in pipelines. When the concentration of the drag reducer was 0.03% in fresh water, drag reduction rate can be up to 76.7%, while in high concentration brine, still as high as 76.2%. It shows that salt has no obvious negative impact on the drag reduction rate. That is also to say, in the case of low viscosity, the viscosity change has no obvious impact on drag reduction rate. From the Cryo-TEM observation, it can be concluded that the drag reducer forms sparse network structures in water, which is the direct reason for drag reducing effect. This finding provides knowledge regarding the development of new drag reducers.

**Supplementary Information:**

The online version contains supplementary material available at 10.1186/s13065-023-00968-5.

## Introduction

All headlines around the world continue to report the crisis of energy shortage [1], especially in the modern industrial society, people are in great demand for oil and natural gas as chemical raw materials, fuels, and national strategic reserves [2]. If the energy supply is insufficient, for example, long-term shortage in coal, oil or natural gas, it will lead to the rise of transportation costs, price, living costs, widening the gap between the rich and the poor, and many other malignant consequences [3]. What matters to everyone, people will be tortured from the cold and many other adverse effects in winter [4]. In previous report, natural gas has become the largest source of fuel for electricity generation in the US, and accounts for a third of energy production and consumption, in addition, the development of oil and gas resources will also provide a large number of job opportunities [5]. For these obvious reasons, the importance of natural gas development is enormous.

Unfortunately, not all countries in the world are rich in oil and gas, or the oil and gas resources are difficult to be developed and have to continue staying underground [6, 7]. In recent years, shale gas is considered to be the most promising clean energy source and has been sought after by people [8, 9]. Generally, shale gas is stored in reservoirs with ultra-low permeability, which is extremely difficult to be exploited or to be developed with low cost. Researchers have made great efforts to solve this problem. In recent years, it has been wildly accepted that hydraulic fracturing stimulation is the most effective way to achieve commercial oil and gas production from low-permeability formations, particularly from unconventional reservoirs, e.g. tight gas, tight oil and shale gas reservoirs [10–12]. The basic principle of hydraulic fracturing is using large amount of fluid (fracturing fluid) to carry proppant (e.g. ceramsite or quartz sand) into the reservoir, forming countless tiny cracks in the dense rock, and then, the liquid flows back, leaving the proppant in the fractures, thus improving the oil and gas seepage area and increasing the oil and gas well production [13–15].

The US has succeeded in large-scale and low-cost development of shale gas, providing a new way for global oil and gas resources development [16]. China follows closely and also has realized the commercial development of shale gas, which has greatly changed China’s energy supply, additionally, it also offers a variety of job opportunities [17]. The development of shale gas has significant social benefits, and should be developed continuously.

As we all know, when fluid flows in the pipelines under high speed, fluid will enter turbulent state, a lot of energy is lost in the pipeline. In field applications, in order to transfer the pressure which provided by the fracturing trucks to the bottom of the well as much as possible, the fluid must have the characteristic of low friction, even under high-speed flow, the pressure loss is kept at a low level [10, 18]. Besides, to avoid abundant foreign substances causing damage to the reservoir, on the premise of meeting low friction performance requirements, engineers want to use as few materials as possible. Of course, this can also reduce costs [19]. The ultimate goal of hydraulic fracturing is to send proppant into the formation, and fracturing fluid is just a medium [20, 21], therefore, engineers hope that the fracturing fluid can not only enter the formation with extremely low permeability easily, but also flow back to the ground after fracturing unhindered. Consequently, low viscosity fluids are always preferred.

In the common reports, surfactant micelles [22, 23], plant gum [24, 25], polymers [26–28] or surfactant and polymer mixture [29, 30] are often used as drag reducers. In the filed application, dry polymer powder is much preferred by people because of its excellent performance in reducing friction, cheap price, convenient for transportation and storage, high salt-tolerance and a great deal of other aspects [31–33]. More importantly, at low polymer concentrations, the polymer aqueous solution (so called slick water) with low viscosity shows excellent drag reduction performance. This is very meaningful. On the one hand, the damage caused by a large amount of chemicals entering the reservoir can be minimized as much as possible; and on the one hand, the cost is also low; thirdly, low viscosity solution is also easy to flow, which is very conducive to enter the reservoir gaps and also flowback. The method of getting more with one stroke is always liked by people, and this is also what we plan to study.

In fact, not all polymers have excellent drag reduction performance, linear polymer is usually better than branched polymer [34]. Common linear polymers including polyoxyethylene ethers (PEG) and partially hydrolyzed polyacrylamide (PHPAM) have been reported as drag reducers. As technology improves by leaps and bounds, hydrophobically associating polyacrylamide (HAPAM) with better properties than traditional PHPAM has been developed over the past decade [35, 36], and researchers all over the world are still continuously developing new products. Usually, HAPAM refers to the modified polyacrylamide with a small amount of hydrophobic groups grafted on the main backbone of the polymer. The most representative hydrophobic monomers including acrylate, long chain alkyl allyl ammonium halide, etc., the number of side chain carbon atoms generally does not exceed 20. In the work of Mao [35], three types of drag reducer were reported, the drag reducer without hydrophobic monomers worked well in fresh water but dissatisfactory in brine, the other two drag reducers with hydrophobic monomers work good in both fresh water and brine. Lai and colleagues synthesized an anionic polymer as drag reducer [37], they studied the drag reduction properties of highly concentrated polymer solutions (0.1 ~ 0.3%), and they stated that when the concentration exceeds a certain value, the efficiency of drag reduction decreases with the increasing of the concentration, that is, high concentration of drag reducer does not bring high drag reduction rate. In Zhou’s work [38], they claimed that the viscoelasticity of slick-water was determined by the microstructure of the drag reducer, which suggested that the network structure and the drag reduction performance were related to the viscoelasticity. In Zhou’s later work [25, 39], they believed high-viscosity slickwater system was more suitable for hydraulic fracturing in tight gas and/or oil reservoir than traditional low viscosity slick water. But Zhou’s viewpoint runs counter to low-cost development, less than a last resort, low viscosity slick water is still the first choice for shale gas development at present.

China is rich in shale gas resource [40] and has made remarkable achievements in shale gas exploration [41]. In recent years, the development of shale gas is running smoothly in Changning-Weiyuan National Shale Gas Development Demonstration Zone [42–45], and large amount of drag reducers are required [46]. In addition, due to the lack of fresh-water resources in some mountain area or saline land, the abundant fracturing flowback water has also to be used in hydraulic fracturing for the purpose of recycling use [47, 48]. The flowback water usually contains high salt concentration, which requires our drag reducers also have good properties in brine, such as drag reduction performance, and viscosity retention [49].

Considering the broad application and research prospects, more related drag reducers are worthy of further study. In the early shale gas development stage in the Sichuan Basin, anionic drag reducers were common used due to lower price and now, it is popular in many oil and gas blocks. For drag reducer manufacturers, in the production process, to reduce safety risks and costs, aqueous solution polymerization method is preferred. Different from common hydrophobic monomers and HAPAM, in this paper, an ultra-long-structured nonionic hydrophobic water-soluble monomer was synthesized and further, an anionic hydrophobically associative polyacrylamide was then synthesized by use only water as solvent, and aimed to be used as drag reducer. In order to facilitate the use and to deal with the high saline flowback water, the drag reducer we synthesized is solid dry powder.

## Experimental section

### Raw materials

Acryloyl chloride, triton X-114 (TX114), 2-acrylamide-2-methylpro panesulfonic acid (AMPS), 2,2-azobis(2-methylpropionamidine) dihydrochloride (V50), deuteroxide (D_2_O), potassium bromide (KBr), with AR grade were all provided by Aladdin Biochemical Technology Co., Ltd (Shanghai, China) and used without further purification. Ethanol, acrylamide (AM), sodium hydroxide (NaOH), triethylamine (TEA), sodium bicarbonate (NaHCO_3_), 35% HCl with AR grade were provided by Kelong Chemical Co., Ltd (Chengdu, China) and used without further purification. Dichloromethane (DCM, Kelong) was redistilled under calcium hydride (CaH_2_, Aladdin) at 40 °C.

### Experimental characterization

Infrared (IR) spectra were obtained on a FT-IR spectrophotometer (Nicolet 380, Thermo Scientific Brand, USA) with KBr disk by transmitting mode, in the wavenumber range of 4000–500 cm^−1^.

NMR spectra were recorded on Bruker AVANCE III 400 MHz spectrometer (Bruker, Switzerland) in D_2_O, and tetramethylsilane (TMS) was used for the internal standard substance.

Kinematic viscosity was measured by using a capillary viscometer with diameter of 0.8 mm. After pouring the sample into the capillary viscometer, place it in a 25 °C water bath for 30 min and then measure the solution outflow time and calculate the viscosity.

Drag reduction rate (DR) was measured using a pipe friction meter provided by the Research Institute of Natural Gas Technology, Petro-China. The basic DR test principle of the test is to use a pump to drive the water to flow in the pipeline at high speed, and test the pressure value at the inlet and outlet of the pipeline, then, the friction can be calculated according to the pressure difference. The tube flow apparatus consisted of a 4.3 m-long smooth stainless-steel tube, with a tube diameter of 8 mm. Flow rate was measured by a turbine flow meter. Pressure was measured by a differential pressure transducer. The circulating pipe system was calibrated by water. The friction of slick water was tested under different concentrations. This single pass testing system was powered by a high-pressure nitrogen gas source. The relationship between pressure drop and flow rate was determined during the test, and then the friction of water and slick-water could be obtained (Eq. 1). With the friction value of fresh water as blank test, and at a given flow rate, the DR can be calculated by Eq. 2 as follows:

1 $$ F = \frac{{P_{1} - P_{2} }}{4.3} $$

2 $$ DR = \frac{{F_{1} - F_{2} }}{{F_{1} }} \times 100\% $$

where *P*_1_ and *P*_2_ are the pressures at inlet and outlet of the pipe respectively (kPa); *F*_1_ is the friction of water and *F*_2_ is the friction of slick-water (kPa∙m^−1^). Compared with our previous work [50], the equipment has been retrofitted and now has a maximum flow rate of 12 m/s.

The average molecular weight ($\overline{Mw}$) of P (AM-AMPS-AT114) was determined by the static light scattering (SLS) method at λ = 532 nm on a BI-200SM laser scattering system (Brookhaven Instrument Co., America). Figure 3 shows the static Zimm plot of P(AM-AMPS-AT114) according to the Zimm equation Eq. 3:

3 $$ \frac{KC}{{R_{\theta } }} = \frac{1}{{\overline{Mw} }}\left[ {1 + \frac{1}{3}\left\langle {R_{g}^{2} } \right\rangle q^{2} } \right] + 2A_{2} C $$

where $$K = 4\pi^{2} (dn/dc)^{2} n_{0}^{2} /N_{A} \lambda_{0}^{4}$$ and $$q = (4\pi n/\lambda_{0} )\sin (\theta /2)$$ with $$N_{A} ,$$$$dn/dc$$, $$n$$, and $$\lambda_{0}$$ being Avogardro number, the specific refractive index increment, the solvent refractive index, and the wavelength of laser light in a vacuum, respectively; and A_2_ is the second virial coefficient. The Zimm equation could be reduced to Eq. 4, the simplest form:

4 $$ \frac{Kc}{{\Delta R\left( {\theta \to 0,c \to 0} \right)}} = \frac{1}{{\overline{Mw} }} $$

Then the $\overline{Mw}$ of P(AM-AMPS-AT114) can be obtained from the inverse of a double extrapolated value to zero angle and zero concentration from many angles and many concentration measurement results.

Cryo-TEM characterization was performed as follows [50]. About 5 μL of liquid sample was placed on a TEM copper grid and moistened with two layers of filter paper, leaving a thin liquid film on the grid. Then, the sample was quickly immersed in a nitrogen-cooled liquid ethane reservoir. The vitrified specimen was then transferred into a JEM2010 cryo-microscope, the observation chamber is kept at low temperature by liquid nitrogen. Finally, digital images were recorded with a Gatan MultiScan charge-coupled device (CCD) camera.

### Synthesis of ultra-long-chain hydrophobic monomer

**Fig. 1:**
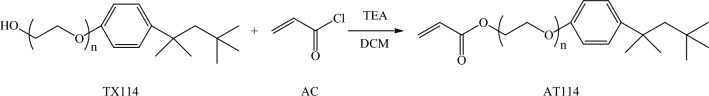
Synthesis route of hydrophobic monomer AT114

The hydrophobic monomer was synthesized as follows [50], with synthetic route shown in Fig. 1. To be specificly, acryloyl chloride (2.2 g, 0.024 mol) was dissolved into 200 mL DCM in a dry beaker and placed in ice-water bath, then, TX114 (11 g, about 0.02 mol) was added dropwise. After 10 min of stirring, TEA (2.66 g, 0.026 mol) was added into the beaker dropwise, white precipitates slowly appeared in the solution. After 6 h, the insoluble matter was eliminated by vacuum filteration, and the filtrate was transferred into a 500 mL separation funne. Then, the filtrate was washed by saturated NaHCO_3_ and 1 wt% HCl sequentially three times. The separated lower layer was then distillated under rotary evaporation. Finally, the ultra-long-chain hydrophobic monomer was obtained as slightly yellowish oily liquid with a yield of 85.2%, named AT114.

### Synthesis of P (AM-AMPS-AT114)

**Fig. 2:**
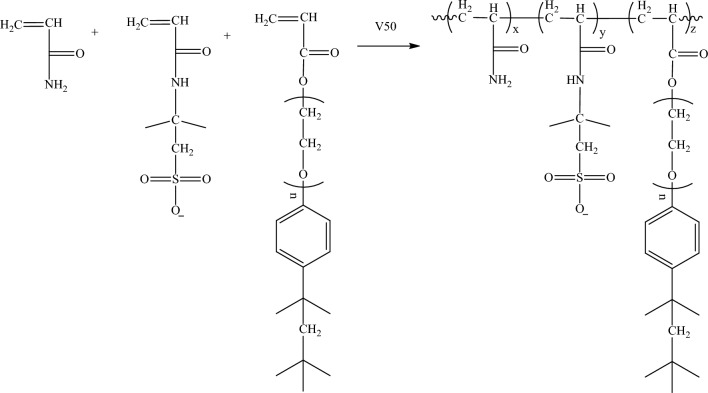
Synthesis route of P (AM-AMPS-AT114)

P (AM-AMPS-AT114) was synthesized via free radical polymerization and aimed to be used as drag reducer, typical synthesis steps are as follows. Firstly, AMPS (2.67 g, 0.0124 mol) was added into a 500 mL beaker and use NaOH (about 0.5 g) solution to adjust pH to 8 ~ 9; secondly, AM (42.6 g, 0.6 mol) and AT114 (1.84 g, 0.003 mol) and water (180 g) were added into the beaker [n(AM): n(AMPS):n(AT100) ≈ 97.5:2:0.5)], after bubbling with N_2_ for 20 min, V-50 (0.05 g) was added under magnetic stirring. The beaker was then sealed and immersed in a 55 °C water bath for 6 h. Thirdly, the jelly-like substance was cut with scissors and washed repeatedly with ethanol. Finally, P (AM-AMPS-AT114) was obtained by drying the white precipitate under 50 °C for 24 h. The synthesis route is shown in Fig. 2.

## Results and discussion

### $\overline{Mw}$ of P (AM-AMPS-AT114)

**Fig. 3:**
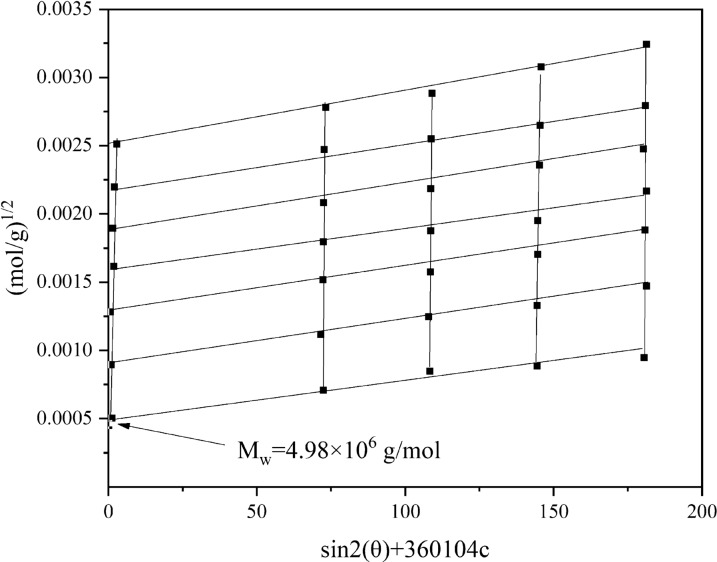
$\overline{Mw}$ of P (AM-AMPS-AT114)

P (AM-AMPS-AT114) aqueous solutions with varies concentrations of 0.05, 0.075, 0.1, 0.2, 0.3, 0.4 and 0.5 mg/mL were firstly prepared, and then each aqueous solutions sample was filtered by 0.45 μm filter, and thirdly, the samples were measured at 135°, 120°, 105°, 90°, 75°, 60°, 45° and 30° by SLS respectively, the result is shown in Fig. 3.

In this work, $\overline{Mw}$ of the P (AM-AMPS-AT114) is about 4.98 × 10^6^ g/mol.

### Drag reduction of P (AM-AMPS-AT114) in fresh water

**Fig. 4:**
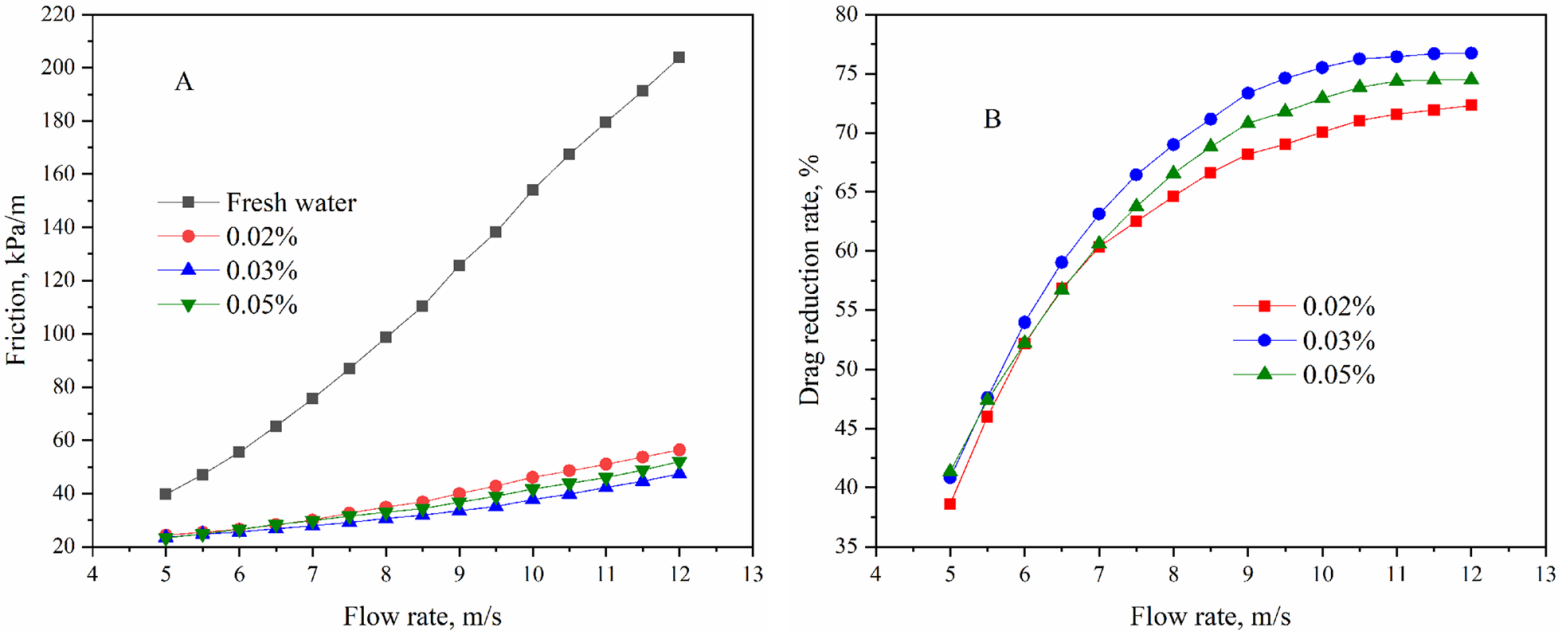
Friction (**A**) and drag reduction rate (**B**) of P (AM-AMPS-AT114) in fresh water

By adding an appropriate amount of P(AM-AMPS-AT114) into 20 L of water, after thoroughly dissolving and stirring, slick-water can be obtained for the friction test. According to the above experimental method, the results are as Fig. 4.

As can be seen in Fig. 4A, when the slick water is injected into the pipe, the friction increases with the increasing flow rate. In the above experimental groups, friction of water is the highest and friction of P (AM-AMPS-AT114) solutions is much lower than that of water. It can also be seen from Fig. 4A that higher drag reducer concentration does not always lead to lower friction, the friction of 0.05% solution is higher than that of 0.03 wt% solution. Using the friction of water as blank, drag reduction rate of slick-water can be obtained as Fig. 4B shown. When the concentration of drag reducer is as low as 0.02%, the maximum drag reduction rate is 72.3%; as the concentration of drag reducer rise to 0.03%, the maximum drag reduction rate is as high as 76.7%; further, when the drag reducer concentration is increased to 0.05%, the maximum drag reduction rate is reduced to 74.5%. Therefore, we can conclude that the higher the concentration of drag reducer did not bring better drag reducing performance. This is similar to previous reports [51]. From the trend of curves change in Fig. 4B, we can reasonably infer that there is an upper limit value for the drag reduction rate, which may be 78% or 80%, or even higher. From Fig. 4B, when the drag reducer concentration is 0.02%, if the flow rate is continued to increase, the drag reduction rate may increase. However, it can be seen from the 0.03% and 0.05% group that if the flow rate is continued to increase, there are almost no changes in drag reduction rate. In previous reports [52], the fluid formed by viscoelastic surfactant also has drag reduction effect, but under high-speed flow, the viscoelastic fluid is shear diluted and loses drag reduction effect. Similarly, maybe at a very high flow rate, the polymer will also be sheared into fragments and lose the drag reduction effect. But under the current laboratory conditions, limited by the power of the equipment and considering safety issues, we were neither able to experimentally confirm our inferred value for the ultimate drag reduction rate, nor to demonstrate that at very high flow rates the polymer would lose drag reduction properties by degrading into fragments. Perhaps in the near future, we can confirm our hypothesis after we upcycle the equipment.

### Drag reduction of P (AM-AMPS-AT114) in brine

In the oil and gas field development industry, fresh water is not always available in some areas with water shortages, for example, mountain areas, salty soils. Formation water with high salt content or fracturing flowback water must be used effectively. In general reports [33, 35], salt can negatively affect the drag reduction rate and viscosity of solutions, even for drag reducers which be claimed to be salt resistant.

**Fig. 5:**
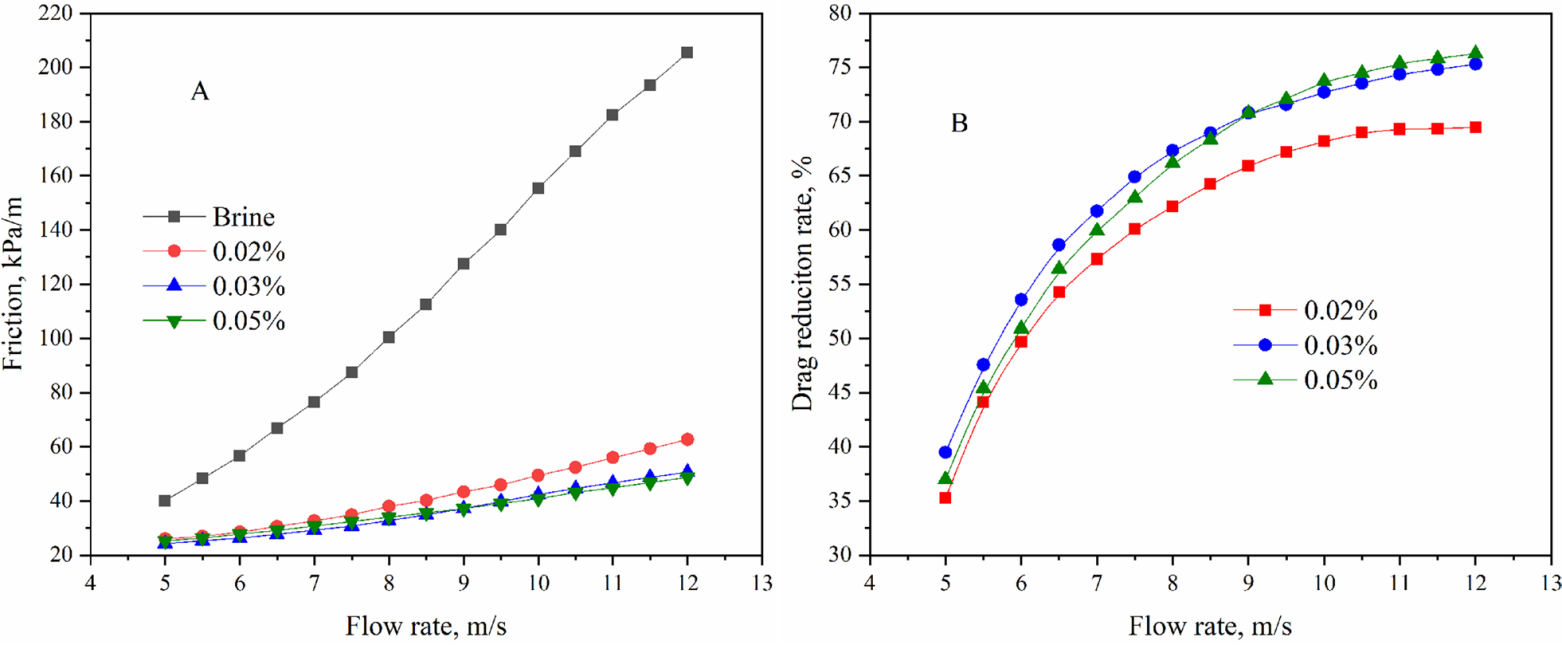
Friction (**A**) and drag reduction rate (**B**) of P (AM-AMPS-AT114) in brine

Here, to simulate the flowback water from shale gas wells in Changning-Weiyuan National Shale Gas Development Demonstration Zone, 20 L brine containing 1000 g of NaCl and 160 g of CaCl_2_ was adopted as simulate flowback water. Then, an amount of P(AM-AMPS-AT114) was added into the brine, further, friction was measured as mentioned above and the results are shown in Fig. 5.

As shown in Fig. 5A, the friction curves of salt water and slick water are extremely similar to Fig. 4A, with the frictions of slick water being much lower than that of salt water. As can be seen from Fig. 5B, when the concentration of drag reducer is 0.02%, the highest drag reduction rate is about 69.5%, lower than that in fresh water; when the concentrations are 0.03% and 0.05%, the highest drag reduction rate are as high as 75.3% and 76.2%, respectively. Compared with Fig. 4B and Fig. 5B, in salt water, higher drag reducer concentrations result in higher drag reduction rate.

### Viscosity comparison

**Fig. 6:**
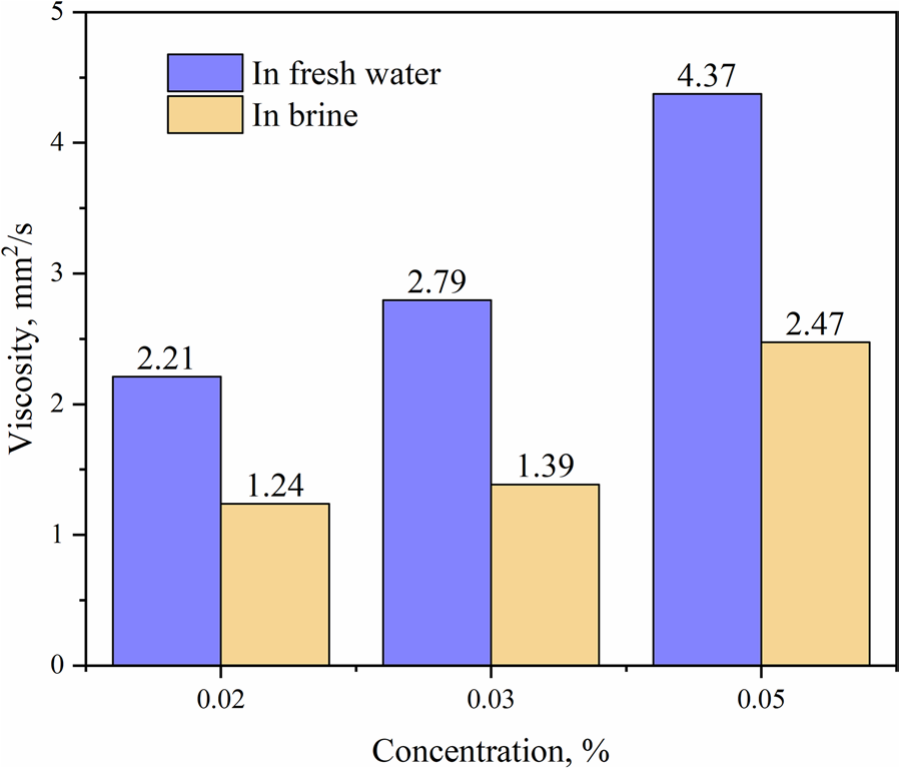
Viscosity of slick water in fresh water and brine

Apart from the effective content of the drag reducer, we believe that the viscosity of slick water also has impact on the drag reduction rate. Although by naked eye observation, we cannot detect significant difference in the viscosity of slick water in clean water and salt water, the viscosity of slick water in brine is significantly lower than in clean water, and the results are shown in Fig. 6. Clearly, referring to Figs. 4 and 5, we can draw the conclusion that when the effective concentration of drag reduer is sufficient, low viscosity is beneficial for drag reduction performance. In our work, the drag reduction rate and viscosity meet the requirements of China industry standards: NB/T 14003.1-2015 regardless of fresh water or salt water.

In addition, low-viscosity slick water has great advantages, it is easy to be pumped and easy to flow back. It can not only provide water resources for next well, but also avoid reservoir damage caused long-term retention of a large amount of water in the reservoir. In a word, when the drag reduction rate reaches the standard, we hope that the viscosity of the slick water as low as possible, that is, add as little drag reducer as possible is prefered, which can not only save costs, but also avoids unpredictable problems caused by excessive chemicals entsering the reservoir.

### Cryo-TEM of slick water in fresh water and brine

**Fig. 7:**
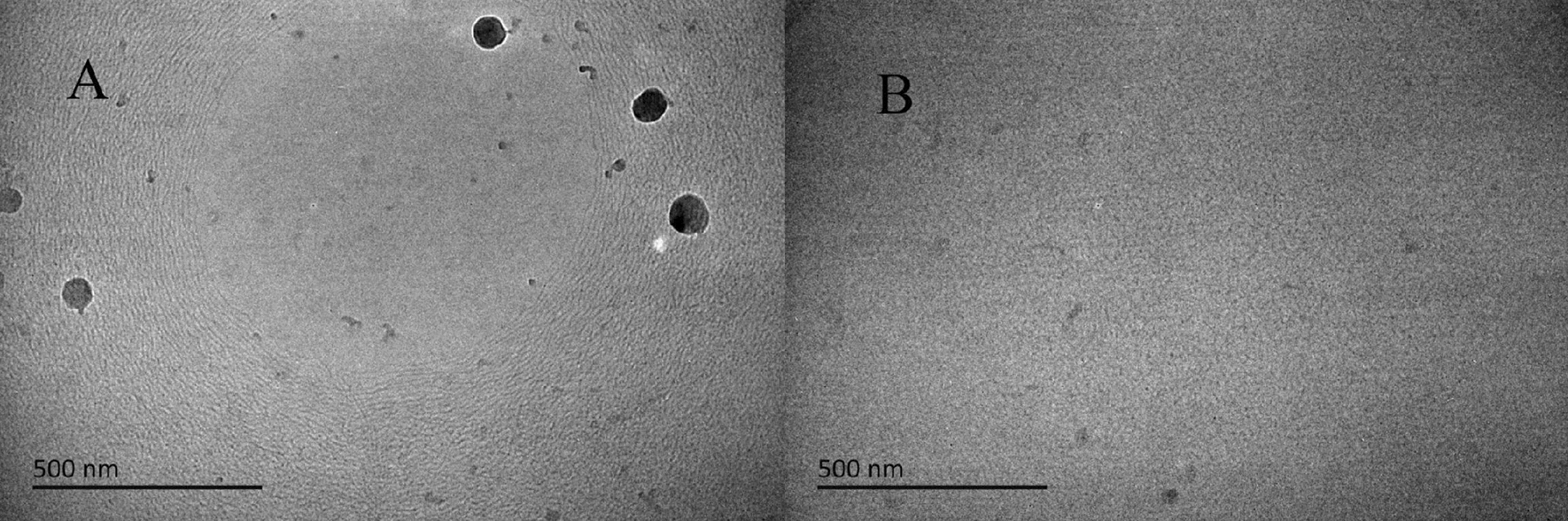
Cryo-TEM of slick water in fresh water and brine

In our previous work [53], we used scanning electron microscopy (SEM) to observe the shape of drag reducer in fresh water and brine, due to the freeze-drying method of sample preparation, active ingredients in slick water, including drag reducer and salt, are left in the mold. Therefore, we cannot observe the real form of drag reducer clearly, it is also possible that only salt crystallization is observed. In contrast, the true state of the solute in solution can be observed using Cryo-TEM and results are shown as Fig. 7.

Dark linear structures can be seen from Fig. 7A, and they are attributed to the formation of network in the solution. Significant black spots are attributed to small particles of polymer powder that are not fully dissolved. A few polymer particles and inconspicuous reticular structure are also both visible in salt water (Fig. 7B), this is because the drag reducer forms a network structure in the solvent, but the network structure in salt water is relatively loose, which is also the reason why the viscosity is lower than that in water.

## Conclusion

A hydrophobically associative polymer with ultra-long side chains was synthesized, it has good drag reduction property and thus has the potential to achieve broad application in hydraulic fracturing in the oil and gas fields, especially in hydraulic fracturing of shale gas or tight oil and gas. Since the nonionic hydrophobic monomer is a surfactant and has good water solubility, we can obtain modified polyacrylamide with excellent performance by using the aqueous solution polymerization method. This method also reduces costs because no oil phase and emulsifier are used. In this experiment, the highest drag reduction rate can reach up to 76.7%, which will help to transfer the power from fracturing trucks to the bottom of the well as much as possible. Low viscosity is conducive to the exertion of drag reduction performance, in brine or flowback fluid, the concentration of drag reducer should be increased appropriately to obtain good drag reduction performance. Cryo-TEM proved that this drag reducer can form network structures in either clear water or salt water.

## Supplementary Information

**Additional file 1.**: Supporting document showing the IR, ^1^H NMR and ^13^C NMR spectra of each compound studied in this paper.

## Data Availability

The datasets used and/or analysed during the current study are available from the corresponding author on reasonable request.
