# Supplementary material for: Synthesis and drag reduction properties of a hydrophobically associative polymer containing ultra-long side chains
Source: BMC Chem. 2023 Jun 5;17(1):48. doi: 10.1186/s13065-023-00968-5 (PMC10240801; doi:10.1186/s13065-023-00968-5)
Supplement: Supplementary file 1 — Additional file 1.: Supporting document showing the IR, 1H NMR and 13C NMR spectra of each compound studied in this paper. [file 13065_2023_968_MOESM1_ESM.doc]

Supplementary Information

**Synthesis and Drag Reduction Properties of a Hydrophobically Associative Polymer Containing Ultra-Long Side Chains**

Xianwu Jing ^1,2 *^, Youquan Liu ^1^, Wanwei Zhao ^1^, Junhong Pu ^3^

**1** Research Institute of Natural Gas Technology, Southwest Oil and Gas Field Company, China National Petroleum Corporation (China) CN, Chengdu, Sichuan, 610213, People’s Republic of China

**2** Shale Gas Evaluation and Exploitation Key Laboratory of Sichuan Province, Sichuan Provincial Department of Science and Technology, Chengdu, Sichuan, 610051, People’s Republic of China

**3** Engineering Technology Department, Southwest Oil and Gas Field Company, China National Petroleum Corporation (China) CN, Chengdu, Sichuan, 610081, People’s Republic of China

*Correspondence: jingxw2018@petrochina.com.cn

## IR and NMR of hydrophobic monomer and drag reducer

IR and NMR are both used to verify whether the alcoholysis reaction of acryloyl chloride and TX114 has been taken place, the IR amd NMR spectrogram are shown as follows.





Fig. S1 IR of TX114, acryloyl chloride and AT114

As shown in Fig. S1, the IR spectra of TX114 and AT114 are very similar, except a remarkable absorption peak at 1730 cm^-1^ in AT114, which is attributed to the stretch vibration of carbonyl in ester group, the difference in infrared spectra proves that the alcoholysis reaction actually occurred. By the way, there is a remarkable absorption peak at 1786 cm^-1^ in acryloyl chloride, which is attributed to the stretch vibration of carbonyl in acryloyl chloride, but the sharp peak at 1786 cm^-1^ cannot be seen in AT114, shows there is no residue of acryloyl chloride in AT114.


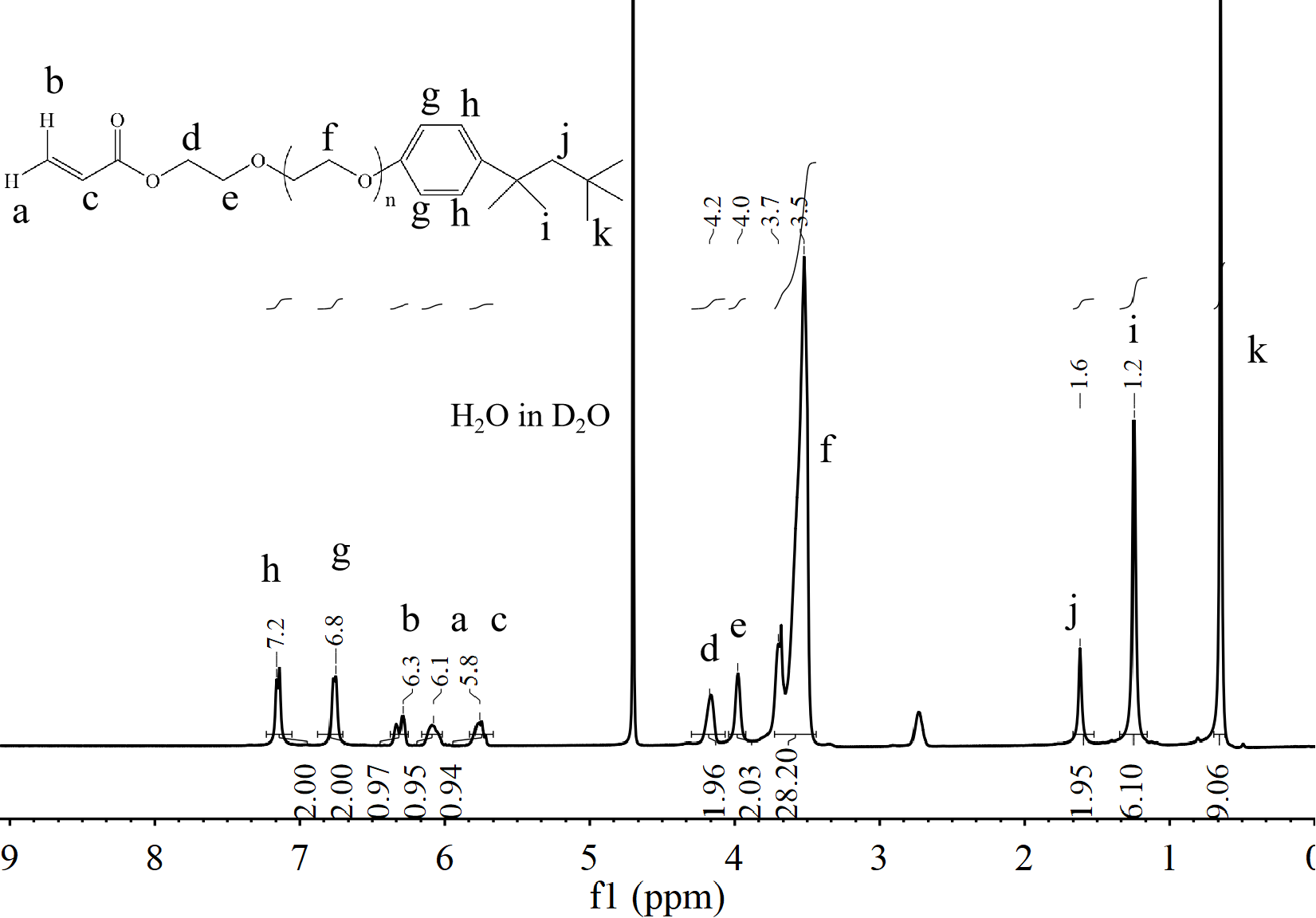


Fig. S2 ^1^H NMR of AT114 (solvent: D_2_O, δ ppm)

^1^H NMR, δ: 7.250~7.028 (d, C***H*** in benzen), 6.858~6.655 (d, C***H*** in benzen), 6.383~6.247 (d, -CH=C***H***_2_), 6.153~5.983 (s, -CH=C***H***_2_), 5.828~5.668 (s, -C***H***=CH_2_), 4.273~4.118 (s, -CH_2_C***H_2_***OC=O), 4.075~3.896 (s, -C***H_2_***CH_2_OC=O), 3.880~3.328 (m, -C***H_2_***C***H_2_***O-), 1.770~1.514 (s, alkyl -CH_2_), 1.463~1.114 (s, alkyl [C***H_3_***]***_2_***), 0.818~0.562 (s, alkyl [C***H_3_***]***_3_***). From the integral area of the ^1^H NMR spectrum, it can be deduced that the purity of AT114 is approximately 100%, but there may also be trace amount of TX114. From the integral area of the peak f, it can be inferred that the number of repeat units of -CH_2_CH_2_O- is 8.


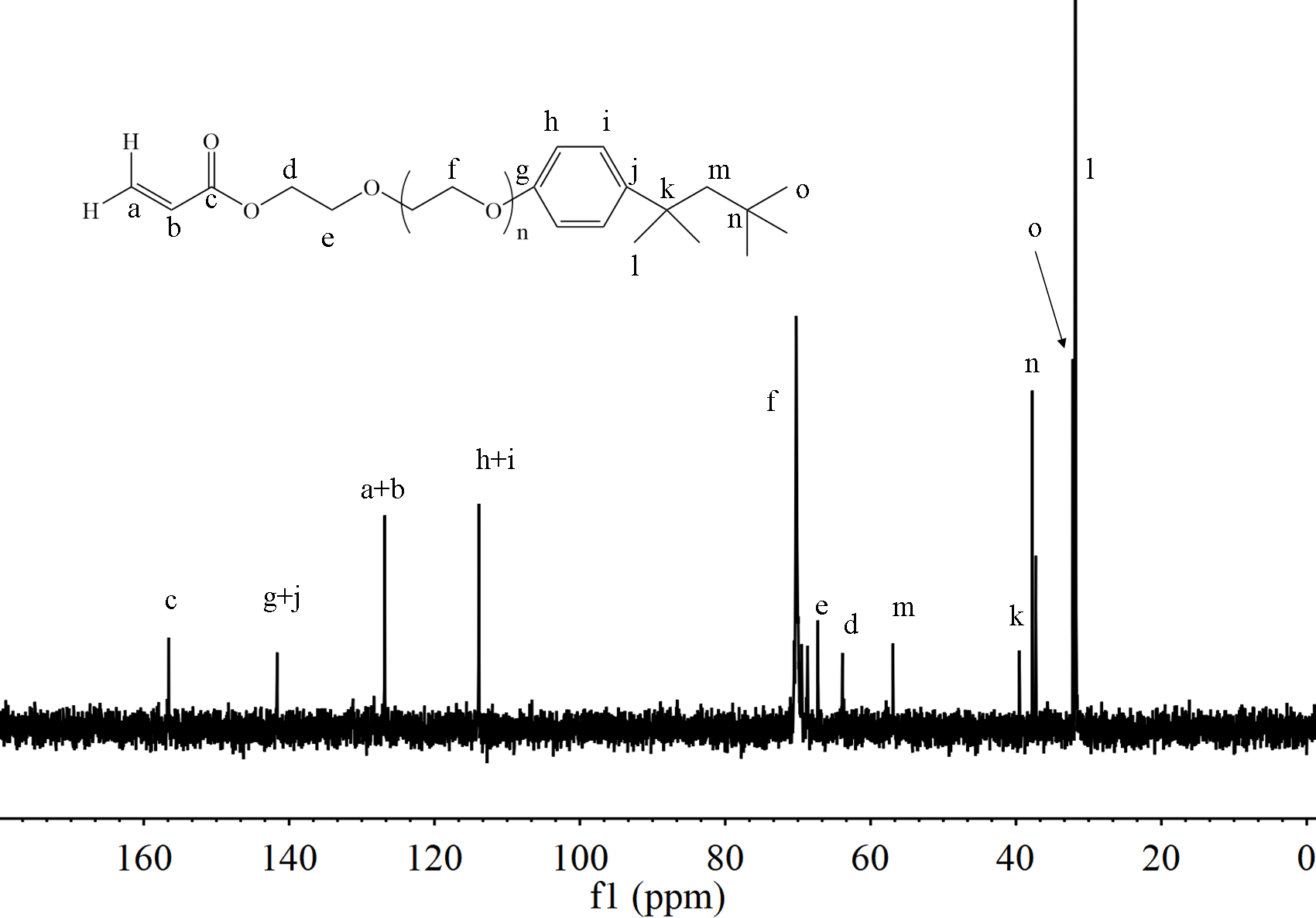


Fig. S3 ^13^C NMR of AT114 (solvent: D_2_O, δ ppm)

^13^C NMR, δ: 156.7 (-***C***=O), 141.8 (-O-***C***H- and -***C***H-C-CH_3_ in benzene), 127.0 (***C***H_2_=***C***H-), 113.9 (-***C***H_2_- in benzene), 70.5 (-***C***H_2_***C***H_2_O-), 67.2 (-***C***H_2_CH_2_OC=O), 63.7(-CH_2_***C***H_2_OC=O), 57.1 (-C-***C***H_2_-C-), 39.6 (-***C***-(CH_3_)_2_), 37.7 (-***C***-(CH_3_)_3_), 32.3 (-C-(***C***H_3_)_3_), 31.7 (-C-(***C***H_3_)_2_).

From the ^1^H NMR and ^13^C NMR spectrum in Fig. S2 and Fig. S3, it is undoubtedly that the slightly yellowish oily liquid is perfect the product what we expected.


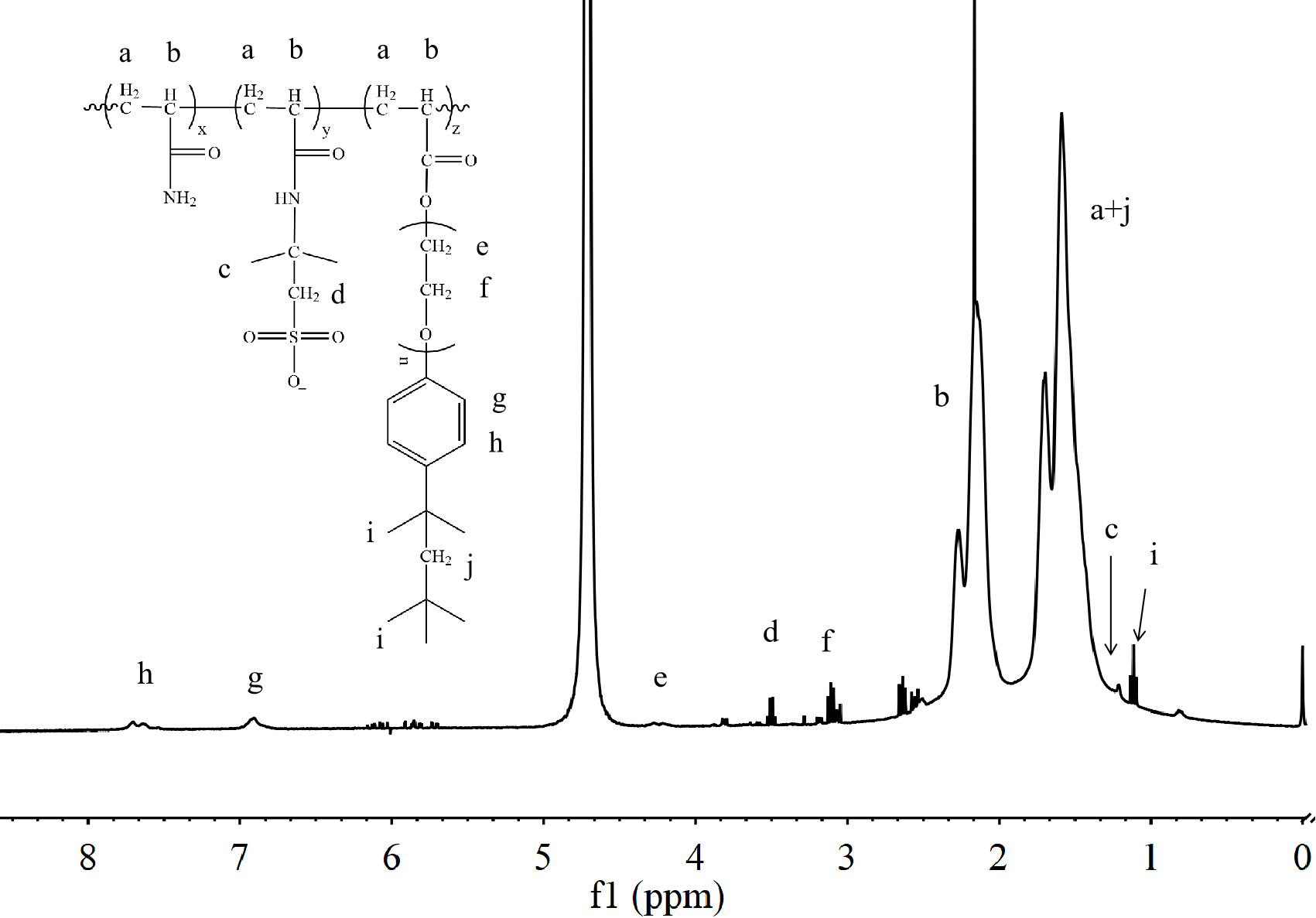


Fig. S4 ^1^H NMR of P(AM-AMPS-AT114) (solvent: D_2_O)

Furthermore, P(AM-AMPS-AT114) was characterized by ^1^H NMR, the result is shown in Fig. S4. ^1^H NMR, δ: 7.746~7.583 (-CH_2_ in benzen), 6.966~6.844 (-CH_2_ in benzen), 4.302~4.180 (-CH_2_C***H_2_***OC=O in AT114), 3.571~3.441 (-HN-C***H_2_***-S- in AMPS), 3.204~ 3.018 (-C***H_2_***C***H_2_***O- in AT114), 2.417~1.977 (-CH_2_-C***H***- in the main chain), 1.921~1.320(-C***H_2_***-CH- in the main chain, -C-C***H***_2_-C- in AT114), 1.286~1.174 (-C-(C***H***_3_)_2_ in AMPS), 1.149~1.052 (-C***H***_3_ in the terminal of AT114).
